# Physical activity for blood glucose control in gestational diabetes mellitus: rationale and recommendations for translational behavioral interventions

**DOI:** 10.1186/s40842-021-00120-z

**Published:** 2021-04-25

**Authors:** Oluwafemifola Onaade, Jill M. Maples, Bethany Rand, Kimberly B. Fortner, Nikki B. Zite, Samantha F. Ehrlich

**Affiliations:** 1 Department of Public Health, The University of Tennessee, 369 HPER, 1914 Andy Holt Ave., TN 37996 Knoxville, USA; 2 Department of Obstetrics and Gynecology, Graduate School of Medicine, The University of Tennessee, Knoxville, TN USA

**Keywords:** Gestational diabetes, Behavioral intervention, Lifestyle counseling, Physical activity, Walking

## Abstract

Gestational Diabetes Mellitus (GDM) is associated with adverse health outcomes during pregnancy and beyond. Previous randomized controlled trials of exercise interventions have demonstrated that exercise, conducted primarily during supervised sessions, improves maternal glycemic control in women with GDM. However, additional research is needed to develop physical activity interventions that are easily implemented in healthcare settings (e.g., recommendations and strategies to increase non-supervised physical activity). This narrative review presents: current physical activity recommendations for pregnancy and women with GDM; the scientific literature to date on physical activity, particularly walking, and blood glucose control in GDM; rationale for physical activity interventions targeting women with GDM that are appropriate for translation to the clinical setting (e.g., lifestyle interventions that include behavioral counseling with a health coach); and the strategies employed by previous, successful lifestyle interventions for pregnant and postpartum women that were based in clinical settings.

Most previous exercise interventions for blood glucose control in women with GDM have included supervised exercise sessions, and will thus be difficult to translate to the health care system. However, lifestyle interventions for weight maintenance (i.e., healthy diet and physical activity) set in the health care system and delivered by health coaches have been successfully implemented in pregnant and postpartum populations. Therefore, we suggest that future trials examine lifestyle interventions that promote unsupervised walking with evidence-based behavioral strategies (e.g., goal setting, monitoring, and feedback) and consider incorporating the use of physical activity tracking devices to support these strategies.

## Background

Gestational Diabetes Mellitus (GDM) is defined as glucose intolerance first recognized during pregnancy [1]. In 2016, the prevalence of GDM in the United States was about 6 % [2], with increases anticipated, given the on-going obesity epidemic [3]. Poorly controlled GDM is associated with several adverse maternal and neonatal outcomes including increased risk of preterm birth [4], cesarean delivery [5], birth injuries [5, 6], macrosomia, hypoglycemia, and hyperbilirubinemia [7]. Long-term sequelae for the offspring include increased risk of childhood obesity [8] and type 2 diabetes [9], thereby extending the transgenerational cycle of obesity and diabetes [9, 10]. Additionally, compared to women whose pregnancies were not complicated by GDM, those with GDM are seven times more likely to develop type 2 diabetes, especially in the first five years postpartum [11, 12]. The estimated national economic cost of GDM for clinical care of both mother and infant in 2017 was about $1.6 billion [13].

Physical activity has been shown to improve glucose control, in part through the acute effects of contraction-mediated glucose uptake into skeletal muscle [14]. Among women with GDM, previous studies have shown that an acute bout of exercise can improve blood glucose control [15–17]. One study found an improvement in postprandial blood glucose following a single bout of low intensity walking [15]. Likewise, in a study by Coe at al., women with GDM completed a supervised walking bout on a treadmill and a bout of sitting and talking, with the condition order randomized, and found improvements in post postprandial glucose with walking [17]. A a study by Avery et al. found lower blood glucose levels following low and moderate intensity aerobic exercise using cycle ergometer [16].

There is evidence that chronic adaptations to maternal physical activity during pregnancy is beneficial to maternal blood glucose regulation and long term metabolic health of offspring [18]. Two meta-analyses found that maternal exercise interventions among women during pregnancy were effective at lowering the odds of developing GDM [19, 20]. Due to the logistic challenges of studying the impact of maternal physical activity on the long term metabolic health of offspring, longitudinal studies have been primarily conducted in rodent models. In a recent review by Kusuyama et al., 11 different rodent model studies investigating the impact of maternal exercise on adult offspring glucose metabolism were identified [18]. Of these, 8 showed that maternal exercise improved adult offspring glucose metabolism, including preventing the development of metabolic disease [18]. If these findings are applicable to humans, then exercise may be used to combat the transgenerational cycle of obesity and diabetes [9, 10].

Evidence-based strategies for improving physical activity and blood glucose control in women with GDM (i.e., lifestyle interventions appropriate for the clinical setting) would be useful for clinicians providing recommendations for this population. This narrative review presents the rationale for: (1) promoting physical activity in women with GDM based on current recommendations, and the scientific literature to date on physical activity interventions for blood glucose control in GDM, (2) future examination of behavioral lifestyle interventions promoting walking that are delivered via health coaching, and thus appropriate for the clinical setting.

### Current recommendations for physical activity during pregnancy

Based on scientific evidence available to date, several professional organizations have published recommendations for physical activity during pregnancy [21–23]. The American College of Obstetricians and Gynecologists’ (ACOG) guidelines state that “most pregnant patients can exercise and there are few maternal medical conditions in which aerobic exercise is absolutely contraindicated” [22]. Both ACOG and the U.S. Department of Health and Human Services (DHHS) [21, 22] recommend that pregnant women engage in at least 150 min of moderate-intensity aerobic activity per week, similar to physical activity guidelines for the general adult population [21]. ACOG specifically suggests that pregnant women should participate in both aerobic and strength conditioning exercises and recommends moderate-intensity exercise for a minimum of 20 to 30 min per day on most or every day of the week, though guidance on strength conditioning exercises is not provided [22]. Recommendations for exercise in pregnancy by the American College of Sports Medicine (ACSM) also closely follows the guidelines for healthy adults (a minimum of 150 min/week of moderate intensity aerobic exercise) [23].

### Recommendations for physical activity in women with GDM

After diagnosis, GDM treatment begins with medical nutrition therapy, physical activity, and, depending on pregestational weight class, weight management [24]. The *Standards of Medical Care in Diabetes* provides guidance on medical nutrition therapy (i.e., provision of an individualized nutrition plan) and weight management (i.e., promote weight gain according to the 2009 Institute of Medicine recommendations [25], but offer no specific advice pertaining to physical activity [24]. Similarly, ACOG acknowledges that physical activity can help lower blood glucose, but provides no explicit recommendations on physical activity for blood glucose control in women with GDM [22]. The DHHS recognizes the role of physical activity in blood glucose control for type 2 diabetes and recommends that adults with type 2 diabetes engage in at least 150 min of moderate to vigorous physical activity and two days of muscle strengthening per week (preferably in bouts spread throughout the week) [21]. However, there are no specific recommendations for women with GDM [21]. Likewise, ACSM guidelines also do not provide explicit exercise recommendations for women with GDM per se, but instead suggest that an exercise routine should be instituted after discussion with a health care provider and individualized based on health condition, symptoms, and fitness level [23].

In 2010, the American Diabetes Association (ADA) and American College of Sports Medicine (ACSM) disagreed as to the quality of the scientific evidence supporting moderate intensity exercise for lowering blood glucose levels in GDM [26]. Both the ADA and ACSM gave the evidence a ‘B’ rating [26]. However, ADA’s rating of ‘B’ indicates that evidence was obtained from ‘well-conducted cohort or case-control studies’ while ACSM’s rating of ‘B’ indicates that evidence was based on limited data from Randomized Controlled trials (RCTs) [26]. More recently, in a 2016 position statement, the ADA suggested that exercise could improve glycemic control and recommended that women with GDM complete 20 to 30 minutes of moderate-intensity exercise on most or all days of the week, with a rating of ‘B’ for the quality of evidence [27] (i.e., again, evidence to support obtained from ‘well-conducted cohort or case-control studies’) [28]. Overall, current physical activity recommendations for women with GDM do not go beyond basic public health recommendations for pregnant and non-pregnant adults alike. Given the added demands of managing GDM during pregnancy, the provision of a more detailed, evidence-based physical activity prescription, in conjunction with support for health behavior change, may be needed to increase physical activity levels and improve health outcomes in this population.

## Methods

**Fig. 1:**
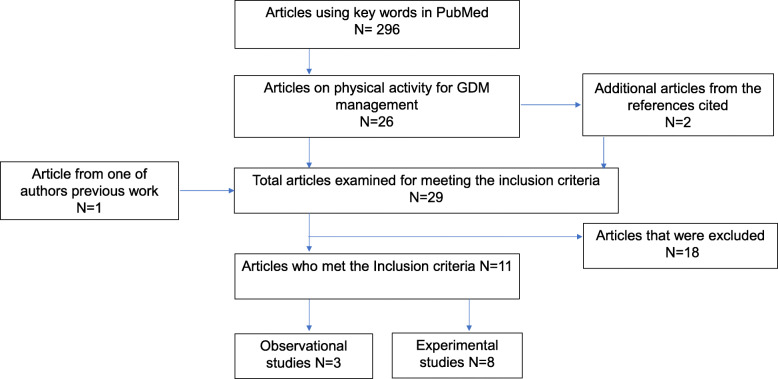
Summary of Literature Search

To review the scientific literature in this area, a primary literature search was independently completed by two authors (OO and BR) using the following keywords in PubMed: “physical activity” AND “exercise” AND “gestational diabetes” AND “treatment” NOT “prevention”. Additional studies were identified by searching all references cited in articles identified in our search. Original research studies were retained for inclusion in this review if: (a) the study population was women with GDM, (b) exposure/intervention was exercise/physical activity (i.e. independent of diet or medication), and (c) the outcome(s) included a measure of glycemic control (fasting blood glucose, postprandial blood glucose, overall glycemic control, pre, and post-exercise blood glucose, random blood glucose and/or insulin requirement). Figure 1 presents a summary of the search. The primary literature search yielded 296 abstracts from which we identified 26 articles that presented data on physical activity for GDM management. Searching the references of these 26 articles identified two additional articles, and one additional article was included from the previous work of one of the co-authors (SE), for a total of 29 articles. Articles were excluded if they were reviews (*N* = 13), controls were not women with GDM (*N* = 1), exercise intervention was acute (*N* = 3) and outcome measures were not related to glucose control (*N* = 1). Eleven studies met our inclusion criteria (*N* = 3 observational [29–31] and *N* = 8 experimental studies [32–39].

## Results

Findings from the observational studies [29–31] suggest that physical activity improves glycemic control in women with gestational diabetes. Evidence from a cohort study of over 900 women with GDM found that moderate-intensity physical activity, as self-reported on the Pregnancy Physical Activity Questionnaire, was significantly associated with optimal glycemic control, defined as ≥ 80 % of self-monitored capillary glucose values meeting clinical targets [30]. In a small study of 24 women with GDM, there was an inverse association between daily steps (objectively assessed by device) and random blood glucose [31]. The authors also reported significantly lower random blood glucose among those who completed ≥ 6000 steps per day [31]. Another small study of 30 women with GDM showed significant improvement in fasting and postprandial glucose levels and lower insulin requirements following participation in low-intensity walking sessions [29].

The results of the experimental studies also suggest that physical activity improves blood glucose control in women with GDM [32–39] (Table 1). It is noteworthy to mention that the outcomes examined in experimental studies varied considerably. Among the experimental studies that found improvement in glycemic control, one measured fasting blood glucose [37], three measured postprandial blood glucose [32, 33, 38], two measured glycated hemoglobin (HbA1c) [32, 37], and two reported insulin requirement [35, 36].

**Table 1:** Characteristics of the experimental studies reviewed

Author	Study Design	Sample size	Intervention condition	Control condition	Outcomes	Findings
**Walking alone**						
Bo et al. 2014 [32] Italy	2 × 2 factorial design, baseline at 24–26 weeks gestation, follow up evaluation at 38th week or before delivery. Four different participant groups: -D: Dietary recommendations only -B: Behavioral dietary recommendations only -E: Exercise only -BE: Behavioral dietary recommendations, and exercise. (Groups E + BE and B + BE were combined for analysis)	200	**Supervision**: No **Frequency**: Every day **Intensity**: 12–14 on the Borg Rating of Perceived Exertion scale **Type**: Walking **Duration**: 20 min/ day or 140 min/week	Behavioral dietary recommendation	*Primary outcome*: Fasting blood glucose *Secondary outcomes*: Postprandial glucose, Hemoglobin A1c (HbA1c), Insulin, Homeostasis-Model-Assessment-Insulin Resistance (HOMAIR)	Adjusted difference in postprandial glucose (mg/dl) for exercise versus control was − 11.1(95 % CI: -16.1, -0.2; *p* < 0.001). Adjusted difference in HbA1c (%) for exercise versus control was − 0.3 (95 % CI: −0.4, − 0.2); *p* < 0.001). No significant difference in Fasting glucose, Log-fasting insulin, and Log-HOMA-IR.
**Walking combined with other Aerobic and/or Resistance Exercises**						
Avery et al. 1997 [34] USA	RCT, enrolled ≤ 34 weeks gestation, follow-up measurements 4-weeks later	29	**Supervision**: Partly **Frequency**: 3–4 times/week till the end of pregnancy **Intensity**: 70 % of calculated maximal heart rate **Type**: Cycle ergometer for supervised, either walking or cycle ergometer for unsupervised (most participants chose walking for unsupervised) **Duration**: 30 min (this included a 5 min warm-up and 5 min cool-down before/after a 20 min session)	Dietary therapy and maintaining current physical activity level.	Hemoglobin A1c, fasting and postprandial blood glucose.	No significant difference in hemoglobin A1c, fasting blood glucose and post prandial glucose.
Halse et al. 2014 [33] Australia	RCT, enrolled 26–30 weeks gestation, follow-up measurements 6-weeks later	40	**Supervision**: Partly, though all exercise occurring at home **Frequency**: 3 supervised and 2 unsupervised per week for approximately 6 weeks **Intensity**: 5 min warm-up at 9–11 RPE (Borg scale) followed by phases of continuous moderate-intensity (12–14 RPE) and interval bouts higher intensity (15–15 RPE) mixed with lower intensity (9–11 RPE). Ends with 5–10 min cool-down at 9–11 RPE **Type**: Upright stationary cycle ergometer for supervised. Not specified for unsupervised sessions, depends on the participant’s choice (walking and cycling were the most commonly selected). **Duration**: Range from 25 to 45 min according to participant ability and progression.	Conventional GDM treatment.	Fasting blood glucose, Postprandial blood glucose, glycosylated hemoglobin and insulin levels	Mean postprandial glucose was significantly lower in exercise group compared with control (*P* = 0.046) No significant difference in daily fasting glucose, HbA1C, fasting glucose and insulin levels after Oral Glucose Tolerance Test.
Sklempe Kokic et al. 2018 [38] Croatia	RCT, enrolled ≤ 30 weeks gestation, follow up data on glucose levels collected monthly /bi-monthly till end of pregnancy (38–40 weeks; data abstracted following childbirth)	42	**Supervision**: Partly **Frequency**: 2 times/ week of supervised session and unsupervised walking daily for a minimum of 6 weeks **Intensity**: 13–14 on the Borg Rating of Perceived Exertion scale for aerobic and resistance exercises parts **Type**: Unsupervised walking plus supervised session which includes aerobic (on treadmill), resistance, pelvic floor and stretching, relaxation **Duration**: 30 min for unsupervised walking, 50–55 min of the supervised session	Standard Antenatal care for GDM	Fasting and postprandial glucose levels at the end of pregnancy	Mean postprandial glucose was lower for exercise group (4.66 ± 0.46 mmol/L) vs. control group (5.30 ± 0.47 mmol/L); (*p* < 0.001) No significant differences in fasting blood glucose.
**Other Aerobic Exercises**						
Bung et al. 1991 [39] USA	RCT, enrolled 27–32 weeks gestation, blood glucose determined weekly thereafter.	41	**Supervision**: Yes **Frequency**: 3 times/week **Intensity**: 50 % VO2 max **Type**: Recumbent bicycle **Duration**: 45 min	Insulin therapy	Mean weekly fasting plasma glucose	No statistical differences between exercise and Insulin subjects were observed in blood glucose measurements.
Jovanovic-Peterson et al. 1989 [37]USA	RCT, Gestational age at enrollment unknown, weekly follow up for 6 weeks	19	**Supervision**: Yes **Frequency**: 3 times/week **Intensity**: Moderate intensity **Type**: Arm ergometer training **Duration**: 20 min for 6 weeks	Diet alone	Fasting glucose levels, post prandial glucose levels, glycosylated hemoglobin	Significant difference in fasting and glycated Hemoglobin.
**Resistance Training**						
deBarros et al. 2010 [36] Brazil	RCT, enrolled sedentary women at 24–34 weeks gestation, followed to end of pregnancy	64	**Supervision**: Partly **Frequency**: 3 times/week **Intensity**: Perceived exertion scale “somewhat heavy” **Description**: Resistance band to target main muscle groups (i.e., stations for chest, back, biceps, triceps, deltoid, quadriceps, thigh, and calf muscles); performed 15 repetitions at each station, with a minimum resting period of 30 s and a maximum of 1 min in between stations. In weeks 1 and 2, underwent 2 circuit series, then 3 circuit series from week 3 to end of pregnancy	Usual prenatal care	Self-monitored capillary glucose values and need for insulin	Exercise group had significantly higher percentage of weeks with 80 % of capillary glucose measurements within target range compared with controls (63 % vs. 41 %). No significant difference in mean glucose levels. Significant decrease in the number of patients who required insulin in exercise group (21.9 %) compared with control group (56.3 %).
Brankston et al. 2004 [35] Canada	RCT, enrolled 26–32 weeks gestation, followed to end of pregnancy	32	**Supervision**: Partly **Frequency**: 3 times/week **Intensity**: felt “somewhat hard” **Description**: Anchored rubber tubing to complete 8 exercises [i.e., plies (squats with outward facing knees), military press, knee extension, hamstring curl, bench press, lateral pull down, seated row, and triceps press] with < 1 min rest in between. Weeks 1 and 2 included 2 sets of 15 repetitions for each exercise; 3 sets of 15 repetitions for each in week 3; 3 sets of 20 repetitions for each from week 4 to end of pregnancy	Standard Diabetic Diet	Primary outcome: insulin requirement Secondary outcome: Latency to insulin treatment and amount of insulin	Significant difference in amount of insulin required (units/kg) (diet alone, 0.48 ± 0.3 versus diet and exercise, 0.22 ± 0.2) Significant difference in latency (weeks) to insulin requirement (diet alone, 1.11 ± 0.8 versus diet and exercise, 3.71 ± 3.1)

The physical activity interventions utilized in the experimental studies reviewed were almost entirely supervised [37, 39] or a combination of supervised and unsupervised exercise sessions [33–35, 38], and thus likely difficult to translate to the health care system and real life situations. We present the characteristics of the eight experimental studies reviewed for this paper in Table 1. Among the exercise interventions for the eight experimental studies identified, two utilized aerobic exercises [37, 39], two utilized resistance exercise [35, 36], three utilized walking in combination with aerobic and/or resistance exercises [33, 34, 38]. Only one study included walking alone and was completely unsupervised [32]. Overall, four of the experimental studies examined the effect of exercise intervention that included some degree of walking [32–34, 38]. Notably, Bo et al. investigated the effects of a completely unsupervised walking intervention in a sample of 200 women with GDM [32]. The authors reported an improvement in postprandial glucose and glycated hemoglobin in response to moderate intensity walking with exercise intervention that included unsupervised moderate intensity walking for 20 min per day (or 140 min/week) [32]. This exercise intervention is unique in that it could easily be prescribed in the clinical setting and achieved in the real-life setting.

## Discussion

### Walking: the preferred physical activity during pregnancy and ideal for GDM management

Physical activity levels in pregnant women are lower than those in non-pregnant populations [40]. The prevalence of pregnant women achieving the higher threshold of ACOG’s 2015 recommendation of ≥ 150 min of physical activity on ≥ 5 days per week, as derived from National Health and Nutrition Examination Survey (NHANES) data between 2007 and 2014 was only approximately 13 % [41]. The intensity and duration of physical activity are typically reduced with the recognition of pregnancy and/or as pregnancy advances [42], and GDM management is limited to the third trimester.

Previous studies have described barriers to physical activity in pregnant women which include lack of time, unfavorable weather, work schedule, childcare, lack of support, and motivation [43–45]. Walking is the most frequently reported modality of physical activity in pregnant women [46]. Walking may offer a potential solution to barriers such as lack of time (e.g., can be integrated into daily activities), childcare responsibilities (e.g., can be done with a stroller or together with children), unfavorable weather (e.g., can be done indoors) and lack of exercise equipment [45]. Three out of the studies that included some degree of walking in the intervention showed promising results to support walking for glycemic control in GDM [32, 33, 38]. The exercise interventions in these studies included unsupervised walking daily for a minimum of 6 weeks [38], unsupervised walking twice a week for about 6 weeks [33], unsupervised walking 20 min in a day or 140 min in a week [32]. There is a paucity of studies examining the effects of behavioral lifestyle interventions promoting unsupervised walking in women with GDM. To improve on current guidelines for GDM management, further investigation of behavioral lifestyle interventions promoting walking is warranted, specifically interventions appropriate for clinical and real life settings [32].

### Future research: interventions appropriate for the health care delivery system

Behavioral interventions delivered via health coaching have successfully been implemented during pregnancy and the postpartum period in the health care delivery system [47–49]. A pilot feasibility RCT that tested a Diabetes Prevention Program (DPP) based lifestyle intervention of diet and physical activity for weight management delivered by trained dieticians demonstrated that such a behavioral lifestyle intervention can be successfully implemented in women with GDM [47]. The findings of a large cluster-randomized trial showed that women with recent GDM who received the DPP-based lifestyle intervention delivered by a health coach (via telephone) were more likely to achieve postpartum weight loss goals [49]. Results from a trial conducted among pregnant women with overweight/obesity reported improvements in gestational weight gain with a similar lifestyle intervention delivered via telehealth [48]. The underlying theory behind these behavioral interventions delivered through health coaching is based on Bandura’s social cognitive theory [50, 51] and the Transtheoretical model [51, 52], that include goal-setting, self-monitoring, and feedback on progress towards behavior goals [51].

Health education and promotion within a ‘coaching’ context show great promise in terms of enhancing well-being and facilitating the achievement of health- or lifestyle-related goals [53, 54]. Individuals trained in health coaching have the professional skills needed to elicit sustainable health-related behavior change by improving patient engagement and activation [54]. The strong argument for the health coaching approach lies in its potential to prompt behavioral changes [54, 55] (such as increasing physical activity) but, importantly and distinctly, the ease of its translation to the health care system [49].

The idea of health coaching for behavior change, particularly in GDM, is not entirely new to the health care system. ADA recommendations for the clinical management of GDM involves medical nutrition therapy, usually delivered by a diabetes educator who is often a registered dietician [24]. Medical nutrition therapy traditionally involves the creation of a personalized nutrition plan as well as monitoring of carbohydrate intake (e.g., counting grams) and feedback/adjustment on this over time to meet clinic targets for capillary glucose values [24]. Based on the theory employed by successful behavioral intervention trials for weight management in pregnancy/postpartum (that included a physical activity component) [47–49, 51], future trials should examine behavioral physical activity interventions for GDM that are delivered by a lifestyle coach and utilize goal setting, monitoring, and feedback.

Given that walking is the most common physical activity modality in pregnant women [40, 45], a behavioral physical activity intervention that promoted walking could use the duration of walking and/or step count achieved during walking to set goals, monitor, and receive feedback over time, similar to goals set for grams of carbohydrate in the context of medical nutrition therapy. Consumer-based wearable devices with the ability to track physical activity (including walking duration and steps) have been shown to increase physical activity levels outside of pregnancy [56]. Trials of behavioral interventions promoting walking for GDM management, with the health behavior change strategy of goal setting potentially supported by wearable technology, are needed to inform the development of a physical activity prescription that can be easily implemented in health care delivery systems.

## Conclusions

In conclusion, scientific evidence strongly suggests that physical activity is beneficial for GDM management. However, further research is needed to identify detailed physical activity prescriptions that are feasible for delivery in the clinical setting and may be integrated into the lifestyle counseling already being utilized for diet. Future research should focus on behavioral physical activity interventions delivered via health coaching and may include the use of physical activity tracking devices to support goal setting, monitoring, and feedback; these strategies are already used in the clinical care of women with GDM.

## Data Availability

Data sharing is not applicable to this article as no datasets were generated or analyzed during the current study.
